# Does simplification signify compromise? Evaluation of different universal adhesives on shear bond strength: *in vitro* study

**DOI:** 10.3389/fbioe.2026.1803547

**Published:** 2026-04-10

**Authors:** Jiyuan Chen, Yuxin Xiao, Yihang Wei, Xinqi Li, Hidehiko Sano, Yuanyuan Kang, Jiale Fu

**Affiliations:** 1 School and Hospital of Stomatology, China Medical University, Shenyang, China; 2 Department of Restorative Dentistry, Division of Oral Health Science, Faculty of Dental Medicine, Hokkaido University, Sapporo, Japan; 3 Department Emergency and Oral Medicine, Liaoning Provincial Key Laboratory of Oral Diseases, School and Hospital of Stomatology, China Medical University, Shenyang, China; 4 Department of Prosthodontics, School and Hospital of Stomatology, China Medical University, Shenyang, China

**Keywords:** light-less, resin cement, shear bond strength (SBS), universal adhesive, zirconia

## Abstract

**Introduction:**

Universal adhesives are widely used in the field of dental prosthetics owing to their broad applicability and clinical convenience. This study aims to evaluate the bonding performance of four adhesives on zirconia and resin material surfaces.

**Methods:**

The shear bond strength (SBS) test results of four adhesives including Single Bond Universal Adhesive (3M1), Scotchbond™ Universal Plus Adhesive (3M2), PALFIQUE UNIVERSAL BOND (TK1) and BONDMER Lightless II (TK2) were studied (n = 10 per adhesive per storage condition) under three storage conditions: constant-temperature water storage at 37 °C for 24 h, 5,000 thermal cycles, and 10,000 thermal cycles. Three-way ANOVA, Tukey HSD test, and Games-Howell test were performed on the outcome data (α = 0.05). The Adhesive Remnant Index was used to evaluate the debonding condition between resin and zirconia surfaces.

**Results:**

All four groups exhibited acceptable bond strength measured at 24 h in a 37 °C constant-temperature water bath and significant differences were observed among the four groups (*p* < 0.05). After thermal cycling, the bond strength of all groups showed significant decline, with only TK1 yielding detectable data. Three-way ANOVA results indicated that all factors—storage conditions (*p* < 0.001), brands (*p* < 0.05), and generations (*p* < 0.05)—exerted significant effects on SBS. At the same time, significant differences were observed between the interaction among the three factors (*p* < 0.001).

**Conclusion:**

The bonding performance of universal adhesives of different generations and brands is material-dependent. After 5,000 thermal cycles, the SBS differed significantly among brands and generations of adhesives, with TK1 exhibiting better performance in this study. Technological advances have introduced user-friendly products with simplified application procedures. However, clinicians should adopt an evidence-based perspective and focus on clinical effect and experimental data of materials.

## 1 Introduction

Currently, owing to high mechanical strength and favorable biocompatibility, zirconia ceramic has been extensively adopted in prosthodontics and is regarded as the first-choice material for dental restorations (Li et al., 2020). However, with conventional bonding protocols, zirconia exhibits inferior bonding performance to silica-based ceramic on account of its surface inertia and poor reactivity (Thompson et al., 2011). Moreover, the conventional cementation protocol for restorations generally involves complex steps, leading to high technique sensitivity and potentially compromising the patient experience (Lima et al., 2023). In 2011, universal adhesives were introduced, featuring simplified steps, versatility across multiple substrates, and applicability for both direct and indirect restorations, thereby significantly streamlining clinical practice. Laboratory reports concerning bond strength to zirconia ceramic and silica-based ceramic have revealed: universal adhesives containing 10-methacryloyloxydecyl dihydrogen phosphate (10-MDP) have been demonstrated to provide reliable bonding performance to zirconia ceramic restorations (Goracci et al., 2022; Lopes et al., 2023; Hu et al., 2022). In clinical practice, the bonding process of silica-based ceramics and zirconia ceramics is different. But for silica-based ceramic, compared to the combination of hydrofluoric acid and silane coupling agent, bonding property and durability of universal adhesives remain significant potential for improvement (Lopes et al., 2023).

At present, in the dental market, most universal adhesives are represented by the single-bottle system while there are also two-bottle clinical products that are applied by mixing Bond A and Bond B in equal proportions. Both of the above two forms of universal adhesives belong to 1-step self-etching system (Arandi, 2023). Single-bottle universal adhesives are light-curable, which is the most common curing mode of adhesives. Camphorquinone (Hasanain and Nassar, 2021) is generally utilized as the initiator and is cured by light irradiation at wave-length of 360–510 nm to initiate the polymerization reaction (Kowalska et al., 2021). Compared to 2- and 3-step systems, although universal adhesive simplifies the application protocols, light-curing operation still occupies chair-side time and cannot be utilized in areas with limited light access (Hirokane et al., 2021). The counterpart is a two-bottle, self-curing system with separated incompatible components. Polymerization of the redox-initiated free radicals is only initiated upon blending the two reactive pastes together. Benzoyl peroxide (BPO)/tertiary aromatic amine is most widely utilized as a chemical initiator (Lamparth et al., 2024). It has been reported that two-bottle universal adhesives provided more durable bonding performance compared with the single-bottle counterparts (Irie et al., 2024).

Universal adhesives are acclaimed multifunctional, providing reliable bonding to major dental substrates including enamel, dentin, ceramics, metals and composite resins (Tsujimoto et al., 2017). Without preparation of specialized primers for each material, universal adhesives are preferred for optimizing clinical operational procedures and consequently reducing chair-side time (Arandi, 2023; Takano et al., 2025). Nevertheless, significant limitations remain: the bond strength is sensitive to shelf-life, storage temperature, number of coating layers, light-curing time, temperature and time of air-blowing (Iliev et al., 2021; Taschner et al., 2014; Mohammed et al., 2023; Saikaew et al., 2019; Marsiglio et al., 2012). Meanwhile, technological sensitivity is still included throughout the application process.

To improve the technical sensitivity of the product and application procedures, dental material manufacturers are upgrading their clinical products, typically including the following methods: adding or replacing functional ingredients, adjusting the concentration of active ingredients, reducing the strict requirements of materials for storage environments, etc. The redeveloped products often attract clinicians’ attention for their distinguishing features compared to previous generations. However, there are rare literature reports on the actual differences between the earlier and current generation products in various application scenes, as well as the comparison of bond strength between adhesives from different manufacturers under equivalent conditions.

Long-term stability of the prosthesis is the primary indicator for evaluating adhesive performance. In current laboratory research, common methods such as constant-temperature water storage and thermal cycling are employed to simulate the temperature variations, component hydrolysis and mechanical stress factors presented in clinical oral environments and during oral functional condition (Lopes et al., 2023; Yang and Liu, 2018; Vasconcelos Monteiro et al., 2022). The adhesives from Solventum (3M) and Tokuyama (TK) are currently widely used in clinical applications. Therefore, in this study, the earlier generation (generation-1) and current generation (generation-2) of universal adhesives from two brands were selected for *in vitro* experiments under different storage conditions to evaluate the effects of brand, generation, and storage condition on the bonding performance between zirconia and resin.

The null hypotheses were as follows: (1) 3M and TK adhesives exhibit equivalent bonding performance under the same storage condition and generation. (2) Generation-1 and generation-2 adhesives from the same brand show no difference in SBS under identical storage conditions. (3) There is no difference in SBS under different storage conditions for adhesives of the same brand and generation.

## 2 Materials and methods

### 2.1 Bonding agents and resin cylinders

**FIGURE 1:**
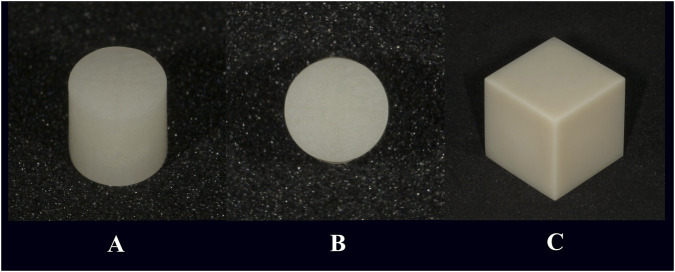
Illustration of the resin cylinder specimens and zirconia specimens. **(A)** Side-view of the resin cylinder; **(B)** Bottom-view of the resin cylinder; **(C)** Side-view of the zirconia cube.

CAD/CAM-produced zirconia cube specimens of Multilayer 3D Pro (24 in total, Aidite Technology, Qinhuangdao, China) with a side length of 2 cm and resin cylinders (216 in total, Aidite Technology, Qinhuangdao, China) with a base radius of 2 mm and a surface area of 12.56 mm^2^ were acquired for the study. The 24 zirconia specimens and 216 resin cylinders were randomly divided into 4 groups, corresponding to the bonding agents, with each group containing 6 zirconia specimens and 54 resin cylinders. The resin cylinder specimens and zirconia specimens are illustrated in Figure 1.

The resin adhesives and resin cements used in this study were as follows:

Group 1 (3M1 + RUC): Single Bond Universal Adhesive + RelyX™ Ultimate Clicker Adhesive Resin Cement;
Group 2 (3M2 + RUC): Scotchbond™ Universal Plus Adhesive + RelyX™ Ultimate Clicker Adhesive Resin Cement;
Group 3 (TK1 + EP): PALFIQUE UNIVERSAL BOND + ESTECEM Plus Universal;
Group 4 (TK2 + EP): BONDMER Lightless II + ESTECEM Plus Universal

The lot number, manufacturers, and detailed chemical composition of the aforementioned bonding agents and the zirconia cleaning paste are presented in Table 1. Application procedures for each product are detailed in Table 2. All operations were conducted at room temperature (25 °C ± 1 °C). The curing modes and storage temperature of four universal adhesives selected in this study are demonstrated in Table 3.

**TABLE 1:** The lot number, manufacturers and chemical formulation of the materials used in present study.

Code	Materials (Lot No.)	Manufacturer	Chemical formulation
3M1	Single Bond Universal Adhesive (4075B)	3M Deutschland GmbH 41453 neuss (Germany)	10-MDP, dimethacrylate resins, HEMA, polyalkenoic acid copolymer, filler, ethanol, water, CQ, γ-MPTS, silane
3M2	Scotchbond™ Universal Plus Adhesive (10860837)		10-MDP, 4,6-dibromoresorcinol-based dimethacrylate diester, DMAE-B, HEMA, acrylic-itaconic copolymer, filler, ethanol, water, CQ, γ-MPTES, APTES, silica
RUC	RelyX™ Ultimate Adhesive Resin Cement (12129322)		Base paste: methacrylate monomers, radiopaque, silanated fillers, initiator components, stabilizers, rheological additives Catalyst paste: methacrylate monomers, radiopaque, alkaline (basic) fillers, initiator components, stabilizers, pigments, rheological additives, fluorescence dye, dual-cure activator for single bond universal adhesive
TK1	PALFIQUE UNIVERSAL BOND (Bond A:133, Bond B:611)	Tokuyama Dental Corp. (Japan)	Bond A: 3D-SR monomer, MTU-6, HEMA, Bis-GMA, TEGDMA, acetone, BHT
			Bond B: γ-MPTES, borate, TMBHP, acetone, isopropyl alcohol, water, BHT
TK2	BONDMER Lightless II (Bond A:056, Bond B:540)		Bond A: phosphate acid, Bis-GMA, MTU-6, HEMA, TEGDMA, acetone
			Bond B: γ-MPTES, acetone, ethanol, water, borate catalyst, peroxide
EP	ESTECEM Plus UNIVERSAL (A087B8)		Bis-GMA, TEGDMA, Bis-MPEPP, silica-zirconia filler, CQ, BPO
IVO	Ivoclean (Z06KJ2)	Ivoclar-vivadent (Liechtenstein)	Zirconium oxide, water, polyethylene glycol, sodium hydroxide, pigments, additives

10-MDP, 10-methacryloyloxydecyl dihydrogen phosphate; HEMA, 2-hydroxyethyl methacrylate; γ-MPTS, γ-methacryloxypropyl trimethoxy silane; DMAE-B, dimethylaminoethanol bitartrate; CQ, camphorquinone; γ-MPTES, γ-mercaptopropyl trimethoxysilane; APTES, 3-aminopropyl triethoxysilane; 3D-SR, monomer, three dimensional self-reinforcing monomer; MTU-6, 6-methacryloxyhexyl 2-thiouracil-5-carboxylate; Bis-GMA, bisphenol-A-diglycidylmethacrylate; TEGDMA, triethylene glycol dimethacrylate; BHT, butylated hydroxytoluene; TMBHP, tert-butyl hydroperoxide; Bis-MPEPP, bisphenol-A-polyethoxy methacrylate; BPO, benzoyl peroxide.

**TABLE 2:** Application procedures of the bonding agents and cleaning paste used in present study.

Code	Application procedures
3M1	1. Apply the adhesive to the zirconia surface and resin cylinders base
	2. Gently blow dry the adhesive for 15 s to a thin uniform coat
	3. Light cure for 20 s
3M2	1. Apply the adhesive to the zirconia surface and resin cylinders base
	2. Gently blow dry the adhesive for 15 s to a thin uniform coat
	3. Light cure for 20 s
TK1	1. Apply 1 drop of liquid A and liquid B into mixing plate. Gently mix A and B together
	2. Apply the TK1 mixture to the zirconia surface and resin cylinders base respectively
	3. Gently air-blow dry the adhesive for 15 s to a thin uniform coat
TK2	1. Apply 1 drop of liquid A and liquid B into the mixing plate. Mix thoroughly with a disposable applicator until the mixed bonding agent turns green
	2. Apply the TK2 mixture to the zirconia surface and resin cylinders base respectively
	3. Gently air-blow dry the adhesive for 15 s to a thin uniform coat
RUC	1. Apply appropriate amount of base paste to the resin cylinders base with syringe
	2. Immediately after applying cement, lightly place the resin cylinder onto zirconia surface
	3. Remove excess cement and light cure for 20 s on each side of the resin cylinder at a distance of approximately 5 mm from the surface
EP	1. Apply appropriate amount paste to the resin cylinders base
	2. Immediately after applying the cement, lightly place the resin cylinder onto zirconia surface
	3. Remove excess cement within 3.5 min and light cure for 20 s on each side of the resin cylinder at a distance of approximately 5 mm from the surface
IVO	1. Cover the entire bonding surface of zirconia with a layer of ivoclean using a microbrush
	2. Apply Ivoclean on zirconia surface for 20 s
	3. Thoroughly rinse with water spray and dry with oil-free air

**TABLE 3:** Curing modes and storage temperature of the four types of universal adhesives.

Universal adhesive	Curing mode	Storage temperature
3M1	Light-cure	2 °C–25 °C
3M2	Light-cure	2 °C–25 °C
TK1	Self-cure	0 °C–10 °C, keep refrigerated and sit until it reaches room temperature before using
TK2	Self-cure	0 °C–25 °C

**FIGURE 2:**
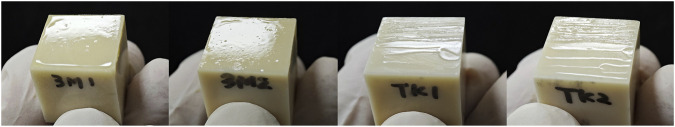
Surface appearance of each universal adhesive applied to zirconia. From left to right: 3M1, 3M2, TK1, TK2; The upper parts of TK1 and TK2 were coated twice.

Figure 2 illustrates the surface appearance of each universal adhesive applied to zirconia.

### 2.2 Surface preparation

The bonding surfaces of zirconia cubes were treated by sandblasting with 50 μm aluminum oxide particles for 20 s at a distance of 2 mm, an angle of 60°, and a pressure of 2.8 bar (Zeighami et al., 2017; Ozcan et al., 2013). Following sandblasting, the bonding surfaces of all zirconia specimens were placed face-down and underwent ultrasonic cleaning in distilled water for 5 min and gentle air-blowing for 15 s subsequently. The application of IVO followed the manufacturer’s instruction: it was applied to the prepared zirconia surface for 20 s, followed by rinsing with distilled water for 15 s and gentle air-blowing.

### 2.3 Application procedure

The resin cylinders were bonded to the zirconia surfaces using a consistent clamp, ensuring that the same pressure (10 N) was applied across all groups. Excess resin cement surrounding the bonding interface was carefully removed by a small brush, taking care not to move or disturb the bonding interface. The procedures for light curing (Kerr Demi Plus, Orange, CA, United States) and the resin cement bonding were described in detail in Table 2.

**FIGURE 3:**
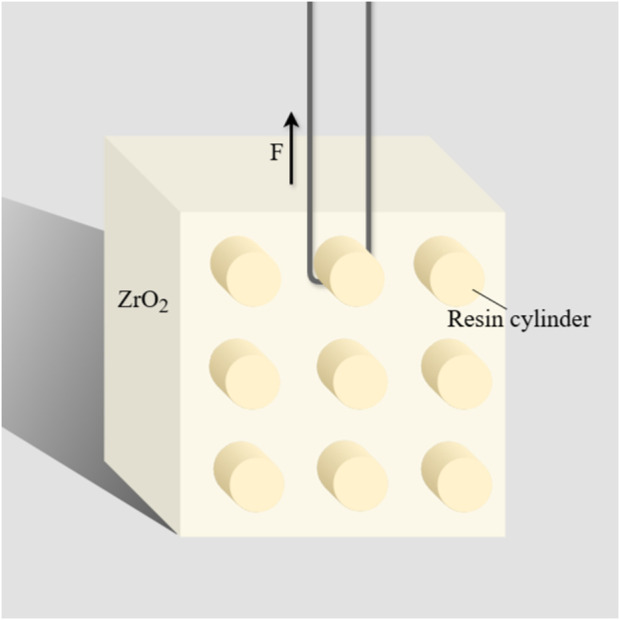
Diagram of specimen setting for SBS test.

A diagram of the bonding of the resin cylinders to the zirconia surface is shown in Figure 3.

### 2.4 Shear bond strength (SBS) test

The resin cylinder specimens bonded with each bonding agent were further divided into three subgroups, with 18 resin cylinder specimens in each subgroup. The three subgroups were assigned to different storage conditions: storage in distilled water at 37 °C for 24 h, 5,000 cycles of thermal cycling, and 10,000 cycles of thermal cycling, respectively. One-third of the resin specimens were tested after storing in an incubator (Shanghai Lichen Bangxi Instrument Technology Co., Ltd., China) with distilled water at 37 °C for 24 h. The remaining specimens underwent thermal cycling in a thermocycler (THE 1400, SD Mechatronik, Germany): one-third underwent 5,000 cycles of thermal cycling between 5 °C and 55 °C, with a dwell time of 25 s per bath and a transfer time of 10 s. One-third underwent 10,000 cycles of thermal cycling between 5 °C and 55 °C, with a dwell time of 25 s per bath and a transfer time of 10 s.

The SBS test was conducted using a universal testing machine (WD-200 Weidu, Wenzhou, China) with a crosshead speed of 1 mm/min (Figure 3). The SBS formula used was P (MPa) = F(N)/S (mm^2^), S = πr^2^, r = 2 mm.

### 2.5 Adhesive remnant index (ARI) score

The ARI scoring was based on digital photographs with the help of a dental digital camera (EyeSpecial C-IV, Shofu, Kyoto, Japan). Following the SBS test, the bonding surfaces of the zirconia and resin specimens were observed. The ARI failure mode was represented by a scale with 5 levels (Score A to Score E) as follows:

Score A: Almost no cement or adhesive remained on the zirconia surface (0%); Score B: 1%–49% of the cement and adhesive remained on the zirconia surface; Score C: 50%–99% of the cement and adhesive remained on the zirconia surface; Score D: Almost all the cement and adhesive remained on the zirconia surface (100%); Score E: The adhesive layer fractured cohesively within the material, leaving remnants on both the zirconia and resin cylinder surfaces.

**FIGURE 4:**
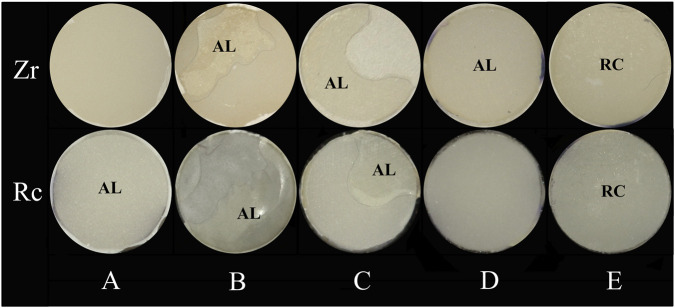
The ARI scores. **(A)** score A 0% on zirconia surface; **(B)** score B 1%–49% on zirconia surface; **(C)** score C 50%–99% on zirconia surface; **(D)** score D 100% on zirconia surface; **(E)** score E on both the zirconia and resin bonding surfaces; Zr: Zirconia; Rc: Resin Cylinder; AL: Adhesive Layer, RC: Resin Cement.

The ARI scoring criteria designated in the present study are shown in Figure 4. To minimize error, the images were scored independently by three calibrated examiners. In cases of disagreement for each specimen, a majority opinion was adopted.

### 2.6 Statistical analysis

Out of 18 results in each group, the highest four and lowest four data points were discarded, and the remaining 10 were considered for analysis (n = 10) (Hu et al., 2022; Su et al., 2025; Hu et al., 2024). The three-way ANOVA (Brands, Generations and Storage Conditions) was used to analyze the influence of these factors on the results. Additionally, multiple comparisons were performed for all groups using either the Tukey HSD test or the Games-Howell test, depending on the homogeneity of variance assumption. This analysis was conducted using SPSS version 27.0, with a significance level of α = 0.05.

### 2.7 Surface evaluation

In this study, the specimens after 5,000 thermal cycles in each group were selected for SEM image acquisition and elemental analysis. Resin cylinder surfaces after debonding were examined with a field emission scanning electron microscopy (FE-SEM) and energy dispersive X-ray spectrometry (EDS; Phenom Pharos G2, Netherlands) to determine elemental composition and distribution.

## 3 Results

### 3.1 Shear bond strength (SBS)

The mean values and standard deviation for each group are summarized in Table 4.

**TABLE 4:** SBS values (MPa) for four bonding agents in three different storage conditions (mean ± SD).

Storage condition	3M1	3M2	TK1	TK2
24 h	4.86 ± 0.49^1,a^	6.99 ± 1.94^1,b^	6.10 ± 0.89^1,b^	4.98 ± 1.16^1,a,b^
5,000 cycles	0.00 ± 0.00^2,a^	0.00 ± 0.00^2,a^	2.96 ± 1.05^2,b^	0.00 ± 0.00^2,a^
10,000 cycles	0.00 ± 0.00^2,a^	0.00 ± 0.00^2,a^	0.16 ± 0.27^3,a^	0.00 ± 0.00^2,a^

The same numbers indicate no significant differences in SBS, within the same bonding agent under different storage conditions (*p* > 0.05).

The same lowercase letters indicate no significant differences in SBS, among different bonding agents under the same storage condition (*p* > 0.05).

Following 24 h storage in 37 °C water, both 3M2 and TK1 exhibited the higher SBS values: 6.99 ± 1.94 MPa and 6.10 ± 0.89 MPa, respectively, without a statistically significant difference (*p* > 0.05). Compared with their brand counterparts, 3M2 and TK1 demonstrated superior SBS. The immediate bond strength of the 3M2 group was significantly higher than that of the 3M1 group (4.86 ± 0.49 MPa, *p* < 0.05), whereas TK1 and TK2 showed comparable values (4.98 ± 1.16 MPa, *p* > 0.05).

After 5,000 thermal cycles, the SBS of all groups showed significant degradation. Only TK1 could be detected (2.96 ± 1.05 MPa) and was significantly higher than the other adhesives (*p* < 0.05).

After 10,000 thermal cycles, no significant difference was observed in SBS of the four adhesives (*p* > 0.05). SBS data could not be measured for the 3M1, 3M2, and TK2 groups. TK1 remained detectable, yielding a residual SBS of 0.16 ± 0.27 MPa.

Three-way ANOVA results (Table 5) revealed that Storage Conditions (*p* < 0.001, F = 627.488), Brands (*p* < 0.05, F = 7.528), and Generations (*p* < 0.05, F = 6.106) all significantly influenced SBS. Significant interactions were found between three factors: Storage Conditions * Brands (*p* < 0.001, F = 15.410), Storage Conditions * Generations (*p* < 0.001, F = 16.970), and Brands * Generations (*p* < 0.001, F = 55.095). Furthermore, notably, the interaction among the three factors—Storage Conditions, Brands, and Generations—also demonstrated significance (*p* < 0.001, F = 11.807).

**TABLE 5:** Three-way ANOVA results of all bonding agents in different storage conditions.

Source	df	Mean square	F	p	Partial Eta squared	Noncent parameter	Observed power
Corrected model	11	78.811	128.371	<0.001	0.929	1412.078	1.000
Intercept	1	565.806	921.607	<0.001	0.895	921.607	1.000
Storage conditions	2	385.236	627.488	<0.001	0.054	6.106	0.688
Brands	1	4.622	7.528	0.007	0.065	7.528	0.776
Generations	1	3.749	6.106	0.015	0.921	1254.976	1.000
Storage conditions * brands	2	9.461	15.410	<0.001	0.338	55.095	1.000
Storage conditions * generations	2	10.418	16.970	<0.001	0.239	33.939	1.000
Brands * generations	1	33.825	55.095	<0.001	0.222	30.820	0.999
Storage conditions * brands* generations	2	7.429	11.807	<0.001	0.179	23.613	0.994

Tables 6–8 present the results of the Games-Howell test and the Tukey HSD test, illustrating the pairwise effects among the three variables.

**TABLE 6:** The mean SBS values (MPa) of the two bonding agent brands (mean ± SD).

Storage condition	3M	TK
24 h	5.93 ± 1.76^1,a^	5.54 ± 1.16^1,a^
5,000 cycles	0.00 ± 0.00^2,a^	1.48 ± 1.68^2,b^
10,000 cycles	0.00 ± 0.00^2,a^	0.08 ± 0.20^3,a^

The same numbers indicate no significant differences in SBS, within the same brand under different storage conditions (*p* > 0.05).

The same lowercase letters indicate no significant differences in SBS, among different brands under the same storage condition (*p* > 0.05).

**TABLE 7:** The mean SBS values (MPa) of the two bonding agent generations (mean ± SD).

Storage condition	Generation1	Generation2
24 h	5.48 ± 0.95^1,a^	5.98 ± 1.87^1,a^
5,000 cycles	1.48 ± 1.68^2,a^	0.00 ± 0.00^2,b^
10,000 cycles	0.08 ± 0.20^3,a^	0.00 ± 0.00^2,a^

The same numbers indicate no significant differences in SBS, within the same generation under different storage conditions (*p* > 0.05).

The same lowercase letters indicate no significant differences in SBS, among different generations under the same storage condition (*p* > 0.05).

**TABLE 8:** The mean SBS values (MPa) of the four bonding agent types (mean ± SD).

Generation	3M	TK
generation1	1.62 ± 2.35^1,a^	3.08 ± 2.59^1,a^
generation2	2.33 ± 3.52^1,a^	1.66 ± 2.47^1,a^

The same numbers indicate no significant differences in SBS, within the same brand of different generations (*p* > 0.05).

The same lowercase letters indicate no significant differences in SBS, among different brands of the same generation (*p* > 0.05).

Table 6 indicated that both the 3M and TK adhesives demonstrate significantly superior performance under 24 h water storage compared to 5,000 and 10,000 thermal cycles later. Under 24 h water storage, 3M was slightly higher than TK, but significant difference was not detected. After 5,000 thermal cycles, the 3M groups totally debonded, while the TK groups retained certain adhesive strength. A significant difference was observed between the two groups (*p* < 0.05). After 5,000 thermal cycles, the 3M groups totally debonded, while the TK groups retained certain adhesive strength. A significant difference was observed between the two groups (*p* > 0.05), though measurable values remained detectable in the TK group.

SBS of both generation-1 and generation-2 adhesives decreased significantly after 5,000 thermal cycles compared to the SBS under 24 h of 37 °C water storage (Table 7). The SBS of generation-1 adhesives was consistently detectable, even remained significantly higher than that of the generation-2 adhesives (*p* < 0.05) post 5,000 thermal cycles, primarily attributed to TK1. After 10,000 thermal cycles, both groups became virtually ineffective.

Integrating the three storage conditions, the SBS ranked respectively as TK1, 3M2, TK2, and 3M1. However, no statistical differences (Table 8, *p* > 0.05) were observed among the four adhesives. SBS value of TK1 demonstrated 3.08 ± 2.59 MPa, slightly higher than the three other bonding agents, attributed to TK1’s superior performance compared to the other groups post thermal cycling.

### 3.2 Failure modes

**FIGURE 5:**
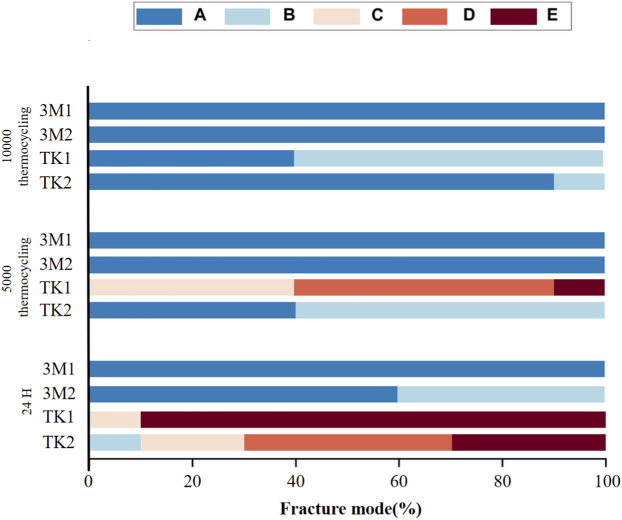
Percentages (%) of the different failure modes after SBS test of resin cylinders. **(A–E)** correspond to Score A to Score E.

The ARI scores for the four adhesives are shown in Table 9 and Figure 5. The two 3M products both exhibited a strong trend to Mode A under all three storage conditions. 3M1 remained at A under all conditions, while 3M2 completely degraded from B to A after thermal cycling. In contrast, TK products performed three distinct modes—C, D, and E—under 24 h conditions. At 24 h, TK1 was mostly concentrated in Mode E. As the number of thermal cycles increased, its failure modes shifted from C and D toward A and B. TK2 showed a similar trend: the initial dispersion of results across score B, C, D, and E gradually diminished, ultimately concentrating in categories A and B post thermal cycling.

**TABLE 9:** ARI scores of each group with different bonding agents bonded to zirconia.

Group tested (24 h/5,000 cycles/10,000 cycles)	ARI scores				
	A	B	C	D	E
3M1	10/10/10	0/0/0	0/0/0	0/0/0	0/0/0
3M2	6/10/10	4/0/0	0/0/0	0/0/0	0/0/0
TK1	0/0/4	0/0/6	1/4/0	0/5/0	9/1/0
TK2	0/4/8	1/6/2	2/0/0	4/0/0	3/0/0

### 3.3 Surface characterization

**FIGURE 6:**
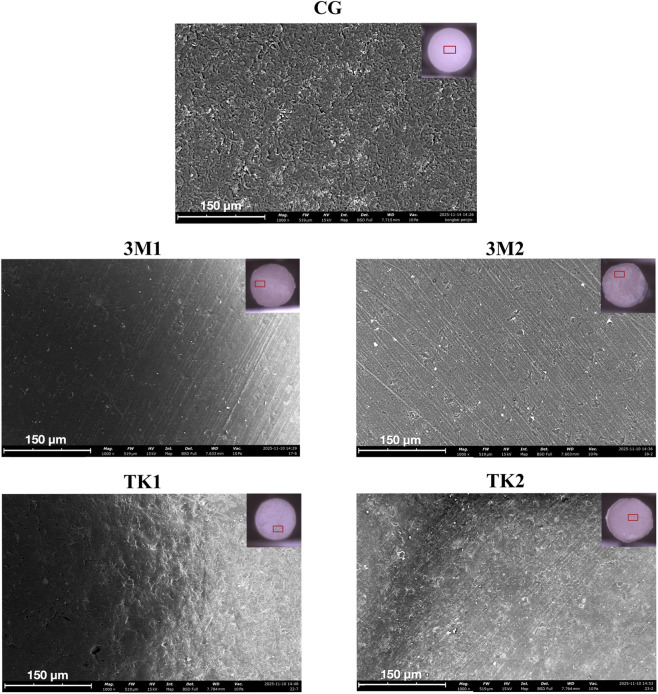
SEM photographs (×1,000 original magnification) of resin cylinder surfaces. CG indicates control group.

**FIGURE 7:**
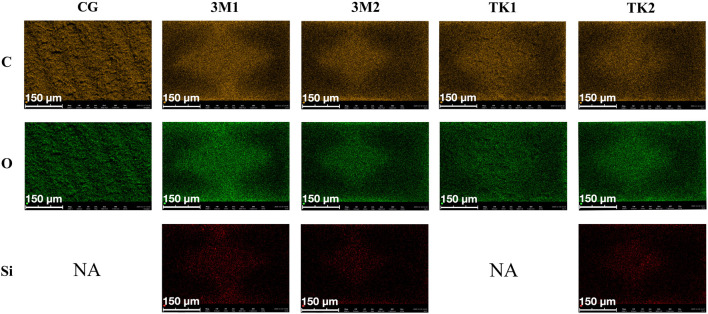
Distribution of elemental composition on the debonding surfaces of resin cylinders (×1,000 original magnification). NA indicates no applicable. CG indicates control group.

Air bubbles in the adhesives could be observed on the surfaces of debonded resin cylinders after 5,000 thermal cycles in groups 3M1, 3M2 and TK2 (Figure 6). The elemental composition and distribution of carbon (C), oxygen (O) and silicon (Si) on the surface of four groups of resin cylinders were shown in Figure 7.

## 4 Discussion

### 4.1 Comparison of bonding agents

This study was aimed at evaluating the impacts of adhesives, from various brands and generations on the bonding performance between zirconia ceramics and resin under different storage conditions. According to Table 6, the bond strength of TK adhesives after 5,000 thermal cycles was significantly higher than that of 3M adhesives (which was undetectable, *p* < 0.05), whereas no significant differences in bond strength were observed under other storage conditions. Therefore, the null hypothesis that 3M and TK adhesives exhibit equivalent bonding performance under the same storage condition and generation could be partially rejected based on these outcomes.

The bonding performance of universal adhesives largely depends on acidic functional monomers (Papadogiannis et al., 2019; Klaisiri et al., 2022). 10-MDP serves as the acidic functional monomer for both 3M adhesives, while the 3D-SR monomer is employed in both TK adhesives (Table 1). 10-MDP is one of the most widely utilized monomers for enhancing the bond strength of zirconia (Llerena-Icochea et al., 2017). 10-MDP facilitates bonding through its bifunctional structure: the hydrophilic phosphate group chemically coordinates with zirconia to form Zr-O-P bonds, whereas the hydrophobic vinyl group ensures integration into the resin matrix through copolymerization (Valente et al., 2020; Ye et al., 2022). The three-dimensional self-reinforcing (3D-SR) monomer, a patented technology by Tokuyama, presents multiple phosphate and polymerizing groups and can form three-dimensional cross-links, thereby eliminating the need for 10-MDP in these two adhesives (Vidal et al., 2024).

RUC, matching both 3M adhesives, contains an integrated activator. Upon contact with the resin cement, the universal adhesives can co-polymerize with the cement, a reaction that compromises adhesion (Aung et al., 2019). Furthermore, the inherently acidic environment within both 3M adhesives can promote hydrolysis and dehydration condensation, leading to chemical instability of silane and a consequential reduction in long-term bond strength (Yao et al., 2018; Yoshihara et al., 2016).

As detailed in Table 7, the generation-1 adhesives exhibited significantly higher SBS compared to undetectable generation-2 adhesives after 5,000 thermal cycles (*p* < 0.05), indicating the intergenerational influence on bonding efficiency. This intergenerational influence was observed only in part of the outcomes, partially rejecting the null hypothesis that generation-1 and generation-2 adhesives from the same brand show no difference in SBS under identical storage conditions.

As a mechanical property indicator, the SBS value is influenced by multiple factors, including the surface geometry of zirconia and resin cylinders, the specimen preparation process, and the configuration of the test fixture (Esmail et al., 2024; Lindemuth and Hagge, 2000). Furthermore, to purely evaluate bonding performance, the surfaces of the zirconia and cylinder specimens used in this study were polished and devoid of retentive features. Consequently, the bond strengths were below the minimum clinical requirement of 10–13 MPa (Lüthy et al., 2006), an outcome attributable to the mechanical testing protocol and experimental design. In clinical settings, the adhesion between zirconia and resin is diverse, including the direct repair of localized chipping of all-ceramic restorations, the adhesive bonding of large-scale fractures, and the attachment of ceramic brackets to resin crown surfaces (Klaisiri et al., 2022). This study demonstrates that thermal cycles induced differential reductions in bond strength among the four adhesive-resin cement combinations, indicating the importance of integrating laboratory evidence into clinical decision.

3M officially claims that when used alone, 3M2 exhibited advanced bonding to glass-ceramics, reaching the gold standard level of traditional, separate silanes^1^ (Thalacker et al., 2024). This breakthrough is attributed to the modified silane coupling agents in 3M2 components (Tannen et al., 2025). γ-MPTES and APTES were used as silane coupling agents and the inorganic phase was synthetic amorphous silica. γ-MPTES and APTES in 3M2 co-hydrolyzed in solvent and condensed with silica to form siloxane bond (Si-O-Si), generating a thick hybrid silane layer (Nishiyama et al., 1987; Maruo et al., 2017). Meanwhile, basic amino groups are generated, neutralizing excess acidic monomers and bonding with resin to enhance the connection between silane and resin (Yao et al., 2021).

This study focused on the evaluation of the bond strength to zirconia. Further studies are required to confirm the bonding performance of 3M2 to silica-based ceramics.

### 4.2 Influence of solvent components

Table 4 demonstrated SBS of four adhesives from various brands and generations under different storage conditions. Unexpectedly, only TK1 exhibited significantly higher SBS than other debonded counterparts (*p* < 0.05). After 10,000 thermal cycles, TK1 maintained detectable values, though decaying to levels showing no statistical difference from other groups. Therefore, considering both bonding strength and durability, TK1 demonstrated superior adhesive performance among the four groups.

Water and organic solvent are both indispensable components of adhesives. Organic solvent acts as a carrier that transports monomers into the collagen interfibrillar spaces (Luque-Martinez et al., 2014), while water is added to trigger the ionization of the respective phosphate monomer (Hiraishi et al., 2005). Within the bonding interface, enhanced bonding strength is thought to be associated with lower solvent concentration and hydrophilicity (Gazal et al., 2024). Hydrolysis is considered as the main reason for bonding failure. Residual solvent was found to inhibit the polymerization of resin and induce deterioration of hybrid layer, thus compromising the clinical performance of adhesives (Chen et al., 2021; Porto et al., 2023). Given that composition and concentration of solvent are critical determinants of adhesive bonding strength (Wang et al., 2007), air-blowing is generally executed in clinical practice to accelerate volatilization and thus reduce residual solvent within the bonding interface.

When ethanol and isopropanol were employed as solvents, they have been suggested to aid in wetting dentin matrix, facilitating the diffusion of resin monomers in collagen network, replacing water from the dentin surface, and providing dry bonding environment (Yiu et al., 2005). The content of hydrophilic monomers has been shown to positively correlate with water absorption of resin polymer network. If hydrophilic monomers are not removed adequately by air-blowing, water absorption of the network may increase, potentially leading to degradation of the adhesive layer. Furthermore, after penetrating the polymer resin chains, the hydrophilic molecules are proposed to act as plasticizers, causing expansion of the network and reducing the friction between polymer chains, thereby softening the material (Zhang et al., 2020).

In this experiment, TK1 exhibited relatively better SBS than TK2 (Table 4). Compared to the refrigeration-requiring TK1, TK2 is allowed to be stored at room temperature (0 °C–25 °C). Additionally, TK2 features a solvent composition change from isopropanol to ethanol, with an increased concentration (from 10%–30% to 25%–35%). The adjustment of solvent concentration and type might expedite the evaporation of solvent under identical air-blowing step, contributing to the differences in thickness and trait of the adhesive layer between TK1 and TK2 (Figure 2). Previous studies have reported that surface heterogeneity (Fu et al., 2012) and excessive thickness (Tang et al., 2024a) of the adhesive layer can lead to decreased adhesion strength. Furthermore, whether room temperature preservation leads to partial evaporation of ethanol requires further validation.

Air-bubbles were observed on the surface of debonded resin cylinders in groups 3M1, 3M2 and TK2 due to solvent evaporation in the adhesive. In contrast, no bubbles were found on the surface of resin cylinders in group TK1 and the control group (Figure 6). The solvents applied in the adhesives of this study were acetone, ethanol, isopropanol, and water. At 25 °C, the vapor pressures decrease in the following sequence: acetone, ethanol, isopropanol, and water (Nist Chemistry WebBook, 2024). The air-bubbles observed in the adhesive layer represent unevaporated water (Ikeda et al., 2008). This finding suggests that TK1 contains less water than the other three adhesives.

### 4.3 The difference between single-bottle and two-bottle universal adhesives

Single-bottle adhesives are generally light-cure systems, and two-bottle bonding agents mainly belong to self-cure system. To meet various requirements, single-bottle adhesives pack all chemical ingredients in a single bottle of agent. Long-term storage in acidic condition for silane coupling agents can lead to emergence of hydrolysis, dehydration and condensation, forming oligomers that cannot bond to glass ceramics. Meantime, acidic functional monomers also undergo hydrolysis, further affecting bonding performance (Naghili et al., 2024). Extra addition of silane coupling agent is necessary for effective bonding to ceramic materials. Material deterioration is avoided fundamentally in two-bottle system by physically isolating incompatible chemical components into separate containers (Ismail et al., 2024), such as segregating silane coupling agents from acidic functional monomers to enhance adhesion to ceramics (Yoshihara et al., 2016).

After thermal cycling, adhesives from TK brand demonstrated higher SBS than products from 3M (Table 6). Among the four adhesives adopted in this study, 3M1 and 3M2 were classified as single-bottle, light-curable adhesives, while TK1 and TK2 belonged to two-bottle, self-cure system. The borate and peroxide in TK1 and TK2 served as a potent chemical polymerization initiator. The borate catalyst reacted with acidic 3D-SR monomers to form boron compounds. The compounds then were oxidized by acidic monomers and generated highly active initiators for chemical polymerization (Kibe et al., 2023). It has been reported that adhesives including borate initiators displayed higher monomer conversion rate than camphorquinone based light-cure adhesives (Vidal et al., 2024), in line with the present study. The data demonstrated that the outstanding performance of TK after thermal cycling may be related to the stability of the initiator and the high monomer conversion rate it generated.

Additionally, 3M2 displayed slightly higher SBS than TK1 after 24 h water bath (Table 4). Nevertheless, a distinct difference in failure mode between these two groups were observed in Table 8. For 3M2, most of the adhesive and cement remained on resin surfaces, whereas cohesive fracture occurred in TK1, likely due to its exceptional adhesion to both zirconia and resin surface. These findings indicate that the diversified composition of these two adhesives determined divergent material property, thereby accounting for the discrepancy in failure mode.

### 4.4 Effect of storage conditions

Thermal stress and hydrolysis induced by thermal cycling between 5 °C and 55 °C simulated the effects of intraoral temperature changes. 10,000 cycles represented approximately 1 year of oral functional status in clinical practice, while 5,000 cycles stood for 6 months (Gale and Darvell, 1999).

SBS declined significantly post thermal cycling (*p* < 0.05). After 5,000 cycles, all four groups showed significant attenuation, and almost completely debonded after 10,000 cycles (Table 4). These results were consistent with the previous study (Moradi et al., 2021). Therefore, the null hypothesis is rejected, which stated that there is no difference in SBS under different storage conditions for adhesives of the same brand and generation.

It could be considered that the rapid decline in SBS post thermal cycling was related to the HEMA component contained in all four adhesives (Table 1). HEMA is a highly hydrophilic functional monomer that can reduce resin viscosity to form a more uniform bonding layer, promote resin infiltration into dentin, and improve water absorption in deeper dentin (Tang et al., 2024b; Abdelkhalek et al., 2025).

However, as previous researches have shown, HEMA may decrease the conversion degree of monomers (Wu et al., 2024; Kitahara et al., 2024) and impair long-term bonding performance due to water absorption and adhesive interface hydrolysis caused by its hydrophilicity (Hu et al., 2024).

### 4.5 Explanation of failure modes

The presence of an adhesive layer can be verified by detecting the Si element, a component of the filler in resin cement (Hu et al., 2022; Hu et al., 2024). In the 3M1, 3M2, and TK2 groups, Si was detected in the residual cement layer on the zirconia surface, whereas it was not detected in the TK1 group or control groups (Figure 7). The adhesive layers of 3M1, 3M2, and TK2 underwent hydrolysis and debonded on the zirconia surface, remaining the adhesive layer on the surface of the resin cylinder. In contrast, TK1 demonstrated better hydrolysis resistance and achieved measurable SBS.

This finding is consistent with the ARI results (Table 9). After 5,000 thermal cycles, most specimens exhibited resin cement and adhesive remnants on the zirconia surface. Following 10,000 thermal cycles, the majority of TK1 specimens still retained a part of resin cement and adhesive on the zirconia surface, whereas no remnants were observed on the zirconia surface in other groups. Therefore, it is inferred that TK1 exhibits superior bonding performance to zirconia than to resin.

Adhesives of two brands exhibited completely different failure modes. Two types of adhesives of 3M showed the trend to remain on the resin surface while almost no residue on the zirconia surface, particularly post thermal cycling. In contrast, TK adhesives tended to remain on the zirconia surface. Interestingly, a special failure mode was observed in some specimens, with adhesive scattered on both the resin and zirconia surfaces (Table 9). This represents cohesive failure, which occurs within the adhesive layer (Ramos et al., 2024; Janson et al., 2025).

Yuta Tsuji analyzed the failure mode of epoxy-hydroxylated layer, proposing a competition between interfacial and cohesive failure. When the maximum force required for interfacial failure exceeded that for cohesive failure, cohesive failure occurred. Otherwise, the interfacial failure occurred (Tsuji, 2024). Therefore, when the chemical interaction on the interface is sufficient, fractures are forced to occur inside the bonding layer, resulting in the cohesive failure of TK adhesives.

### 4.6 Consideration of chair-side convenience and clinical bonding performance

The four universal adhesives selected in this study have certain differences in curing modes and storage temperature variations, representing varying degrees of clinical convenience, as shown in Table 3. TK1 is a self-cure adhesive without light-cure step, simplifying the chair-side procedures. But its obligatory requirement of refrigerated storage increases the complexity of material storage to a certain extent. TK2 changes the storage conditions to room temperature with non-essential light-cure step, which is more clinically convenient among the four groups. In contrast, the two universal adhesives from 3M company both require the light-cure step, owning the same operational procedures of All-in-1 system. The Tokuyama corporation incorporated a novel initiator system to omit the light-cure process of the conventional “applying—air-blowing—light-curing” protocol. One of the distinctions between the two generations lies in the broadened storage temperature range of TK2, with no more requirement for refrigeration.

Refrigerated storage is necessary to preserve efficacy of resin-based products (Sabbagh et al., 2018), especially in summer or in regions of elevated ambient temperature. As demonstrated in Table 3, it is evident that TK1 revealed higher SBS among all groups under different storage conditions. While TK2, without refrigeration, exhibited slightly weaker SBS post thermal cycling, seemingly indicating in this experiment the simplification of operation made concessions in terms of bonding strength. However, 3M adhesives with more complex chair-side operation contrarily showed weaker SBS than TK1 post thermal cycling (Table 8), manifesting that application complexity could not define the adhesive property of materials, which were not contradictory.

The core logic of bonding technology is that greater operational complexity often signifies stronger bond strength. Otherwise, the complexity would be meaningless. Meanwhile, complexity also leads to increased technique sensitivity, such as dentin wet bonding techniques. Accordingly, manufacturers have embarked on the path of simplification: reducing clinical steps in an attempt to reduce technique sensitivity, while accepting compromise in a certain degree. Nevertheless, simplification and novelty should not be achieved at the expense of material properties (Cadenaro et al., 2023).

But does simplification signify compromise? Not necessarily. Technological advancement is the only solution to break through this paradox.

### 4.7 Discussion limitations

This *in vitro* study simulated oral functional condition via thermal cycling, lacking actual temperature of oral cavity and salivary immersion of specimens. Surface treatment solely encompassed sandblasting and cleaning of zirconia and resin cylinder bonding surface, without exploration of zirconia surface modification. Smooth and flat zirconia ceramic surfaces were employed as bonding interfaces in this study. Subsequent researches should include evaluation of various surface treatment methods of zirconia and explore in depth the best-matched pretreatment for each bonding agent, hereby to render instruction for clinical decisions.

Power analysis indicated that the statistical power for Generations and interaction effects was sufficiently high (>0.99). However, for factors such as Storage Conditions and Brands, which had relatively small effect sizes (f < 0.3), the statistical power was slightly below the conventional standard of 0.80. Future studies with larger sample sizes would help improve the statistical power to detect small effects.

## 5 Conclusion

1. The bond strength of universal adhesives of different generations and brands is material-dependent.
2. After 5,000 thermal cycles, the SBS differed significantly among brands and generations of adhesives, with TK1 exhibiting better performance in this study.
3. Technological advances have introduced user-friendly products with simplified application procedures. However, clinicians should adopt an evidence-based perspective and focus on clinical effect and experimental data of materials.

## Data Availability

The raw data supporting the conclusions of this article will be made available by the authors, without undue reservation.

## Footnotes

1
https://multimedia.3m.com/mws/media/1890592O/technical-product-profile-3m-scotchbond-universal-plus-adhesive-int.pdf
